# Ebola and Indirect Effects on Health Service Function in Sierra Leone

**DOI:** 10.1371/currents.outbreaks.0307d588df619f9c9447f8ead5b72b2d

**Published:** 2014-12-19

**Authors:** Håkon Angell Bolkan, Donald Alpha Bash-Taqi, Mohammed Samai, Martin Gerdin, Johan von Schreeb

**Affiliations:** Department of Cancer Research and Molecular Medicine, Norwegian University of Science and Technology, Trondheim, Norway; CapaCare, Trondheim, Norway; Ministry of Health and Sanitation, Freetown, Sierra Leone; College of Medicine and Allied Health Sciences, University of Sierra Leone, Freetown, Sierra Leone; Department of Public Health Sciences, Health system and policy, Karolinska Institute, Stockholm, Sweden; Department of Public Health Sciences, Health system and policy, Karolinska Institute, Stockholm, Sweden

**Keywords:** ebola, Epidemiology, Surveillance

## Abstract

Background: The indirect effects of the Ebola epidemic on health service function may be significant but is not known. The aim of this study was to quantify to what extent admission rates and surgery has changed at health facilities providing such care in Sierra Leone during the time of the Ebola epidemic. Methods: Weekly data on facility inpatient admissions and surgery from admission and surgical theatre register books were retrospectively retrieved during September and October. 21 Community Health Officers enrolled in a surgical task-shifting program personally visited the facilities. The study period was January 6 (week 2) to October 12, (week 41) 2014. Results: Data was retrieved from 40 out of 55 facilities. A total of 62,257 admissions and 12,124 major surgeries were registered for the study period. Total admissions in the week of the first Ebola case were 2,006, median 40 (IQR 20-76) compared to 883, median 12 (IQR 4-30) on the last week of the study. This equals a 70% drop in median number of admissions (p=0.005) between May and October. Total number of major surgeries fell from 342, median 6 (IQR 2-14) to 231, median 3 (IQR 0-6) in the same period, equal 50% reduction in median number of major surgeries (p=0.014). Conclusions: Inpatient health services have been severely affected by the Ebola outbreak. The dramatic documented decline in facility inpatient admissions and major surgery is likely to be an underestimation. Reestablishing such care is urgent and must be a priority.

## Introduction

Ebola Viral Disease (EVD) continues to ravage Sierra Leone. Experts have expressed concerns of complete breakdown in civic society.^1^ To date the direct effects of EVD in Sierra Leone includes over 8,000 infected and 1,820 deaths among them 106 health care workers.^2^ Of the 124 medical doctors in the country, 10 have died due to Ebola, corresponding to a risk ratio of 280 compared to the general population.^2^^,^ 3

In addition to the direct health effects of the viral disease, indirect EVD effects on health service may lead to increased morbidity and mortality. Factors such as patient’s fear of Ebola and death of health care staff has reportedly affected health-seeking behavior and reduced the function of health service.^4^ Patients suffering from complicated deliveries, injuries, incarcerated hernias and malaria may today not find the care they acutely need.^4^^,^ 5

The scale of such indirect effects on health facility function is unknown. Such data remain essential for any health agencies aiming at providing care in the EVD affected country. The aim of this study was to, in the midst of the EVD epidemic, assess to what extent admission rates and surgery has changed in Sierra Leone since the onset of the EVD epidemic.

## Material and Methods

This study was carried out to provide data and guidance for agencies providing health care in Sierra Leone. The study was done in collaboration between the Ministry of Health and Sanitation in Sierra Leone, Karolinska Institutet in Sweden, Norwegian University of Science and Technology and the Non Governmental Organization CapaCare, as part of a new surveillance initiative to monitor effects of the Ebola epidemic on health services. The director of research and Non-Communicable diseases (MS) and the Director of Laboratory and Hospitals (DB-T) of the Ministry of Health and Sanitation both approved the study. Since September 2014 we survey 61 governmental, private non- and for-profit healthcare facilities that offer in-patient care and major surgery. This represents all facilities that offer such care in Sierra Leone identified in a 2013 countrywide study that systematically mapped such facilities.^6^ The study did not include facilities providing inpatient tuberculosis or psychiatric care. For this study six facilities were excluded. Three because they only performed cataract surgery and three were closed in the whole of 2014. Hence 55 facilities were included. A pre-condition for the study was that data collection had to be simple, quick and not expose data collectors to EVD during data collection. We therefore decided to base the study on readily available facility data. We assumed that the number of general inpatient admissions and number of surgeries could serve as proxy indicators on the function of the health facility providing such service.

This study was done with the help of 21 courageous data collectors, Community Health Officers enrolled in a surgical task-shifting program. They were trained in data collection during two days and received a tablet with SIM card to allow direct data entering. They personally visited the facilities.^7^ Weekly data on facility inpatient admissions and surgery from 2014 from admission and surgical theatre register books were retrospectively retrieved during September and October. The Ministry of Health and Sanitation wrote a letter encouraging all healthcare facilities share the required data, which enabled data collectors to access the facility registry books. Data was entered into excel, compiled and analyzed with Stata®. We used a 5% significance level. Averages are reported as medians with interquartile ranges. Tests were parametric or non-parametric. We used segmented linear regression to detect significant temporal trends.^8^

The study period was January 6 (week 2) to October 12, (week 41) 2014. As the first case of Ebola in Sierra Leone were reported on May 23, we defined the pre-outbreak period as the time between week 2 and week 21 and post-outbreak as week 22-41.

## Results

Out of the 55 facilities included, data was not available for 15; three were not accessible while 12 were closed during our visits, reportedly due to the EVD outbreak. However, it was not possible to get exact information regarding the reasons for their closure. From the remaining 40 facilities, a total of 62,257 admissions and 12,124 major surgeries were registered for the study period.

Out of these, 35,609 admissions were in the period before the outbreak and 26,648 admissions were after the outbreak. Furthermore, 12,124 major surgeries were registered for the study period, out of which 7,078 surgeries were in the period before the outbreak and 5,096 surgeries were after the outbreak.

Total number of admissions in the 40 facilities in week 21, the week of the first Ebola case was 2,006, median per facility was 40 (IQR 20-76). Total number of admissions in week 41, the last week of this study, was 883, median 12 (IQR 4-30) (figure 1). This equals a 70% drop in median number of admissions (p-value=0.005)

**Median (A) and total number (B) of admissions and median (C) and total number (D) of surgeries by week in Sierra Leone until October 12 (week 41), 2014 compared to Ebola cases (WHO data).**
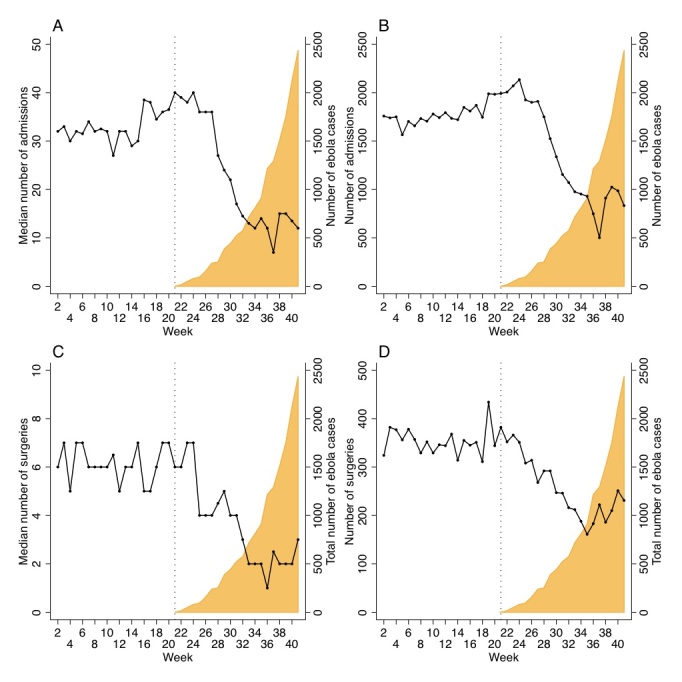

Total number of major surgeries in week 21 was 342, median 6 (IQR 2-14). Total number of major surgeries in week 41 was 231, median 3 (IQR 0-6). This equals a 50% drop in median number of major surgeries (p-value=0.014).

Using segmented linear regression, we estimate that each facility admitted 335 (95% CI 174-536) less patients post-outbreak compared to pre-outbreak (p-value for trend <0.001). Each facility performed 48 (95% CI 16-86) less major surgeries in the post-outbreak period (p-value for trend <0.001).

## Discussion

This is the first study to document the indirect effects of the Ebola epidemic on health facility functioning in Sierra Leone, one of the countries most severely affected by the epidemic. The dramatic documented decline in facility inpatient admissions and major surgery is likely to be an underestimation for several reasons.

First, admissions normally increase due to more malaria during the rainy season, which starts in May. Second, by comparing medians of only facilities with complete data before and after the outbreak, the significance of the post- outbreak-closed health facilities remain unaccounted for. Third, it is likely that patients who normally seek care at the now closed facilitates went to those open, limiting the observed decline.

This study has several limitations. Data is retrospective and based on routine data collected at the facility. We could not document the reasons for why facilities were closed, other than it was because of the Ebola epidemic. Nevertheless we are convinced that the trends identified represent a real decline in facility function. We will continue to monitor the trend over time even when the last case of Ebola has been reported.

The study was conducted in the midst of the epidemic in an extremely challenging context. We were shocked as one of the data collectors fell ill and died of Ebola during the study period. He did however not contract EVD during data collection, but was exposed to EVD as frontline healthcare worker at a governmental hospital.

To put a number on Ebola’s indirect health service effects, our results indicate that an estimated 35,000 sick in Sierra Leone will be excluded from inpatient care from the onset of the epidemic in mid-May and until the end of the year if current low level of admissions remains. Significant international efforts are needed, not only to stop and contain the Ebola epidemic, but also to limit its indirect effects on health service functioning.

## Competing Interests

The authors have declared that no competing interests exist.

## Correspondence

Håkon Angell Bolkan, MD

Norwegian University of Science and Technology

Department of Cancer Research and Molecular Medicine  

Post box 8905 

N-7491 Trondheim,  Norway

Phone: +47 99150768

E-mail: hakon.bolkan@capacare.org
